# The Impact of Secure Messaging in the Treatment of Patients With Diabetes Within a Primary Care Setting: Protocol for a Scoping Review

**DOI:** 10.2196/42339

**Published:** 2023-05-02

**Authors:** Abdul Lawal, Devidas Menon, Ewan Affleck, Tania Stafinski

**Affiliations:** 1 School of Public Health University of Alberta Edmonton, AB Canada; 2 College of Physicians & Surgeons of Alberta Edmonton, AB Canada

**Keywords:** secure messaging, secure message, text messaging, security, privacy, chronic disease, chronic condition, primary care, health outcome, diabetic, diabetes, virtual care, scoping review, review methodology, health care system

## Abstract

**Background:**

Diabetes—a high-burden chronic disease—requires lifetime active management involving the use of different tools and health care resources to improve patient health outcomes. Recent studies have demonstrated promising results regarding the impact of the use of virtual care technology on the treatment of chronic diseases, such as diabetes. However, it is unclear whether the use of technologies, such as secure messaging, improves the quality of care and reduces diabetes-related costs to the health care system.

**Objective:**

The purpose of our scoping review is to explore what is known about the use of secure messaging in the treatment of diabetes within the primary care setting and how its impact has been assessed from the patient and health system perspectives. Our review aims to understand to what extent secure messaging improves the quality of diabetes care.

**Methods:**

Our scoping review will follow the 6-step Arksey and O’Malley methodological framework, as well as the Joanna Briggs Institute methodology for scoping reviews and their recommended tools. The tools to guide the development and reporting of the review in a structured way will include the Population, Concept, and Context framework and the PRISMA-ScR (Preferred Reporting Items for Systematic Reviews and Meta-Analyses Extension for Scoping Reviews) guidelines and checklist. The search strategy was developed iteratively in collaboration with a professional information specialist. Furthermore, a peer review of electronic search strategies was also conducted by an independent, third-party, professional information specialist. A systematic literature search will be conducted against databases, including Ovid MEDLINE ALL, Embase, APA PsycINFO, Cochrane Library on Wiley, CINAHL on EBSCO, and PubMed. Grey literature sources will also be searched for relevant literature. Literature on the use of secure messaging in the treatment of diabetes (types 1 and 2) within a primary care setting will be included. Two reviewers will review the literature based on the inclusion criteria in the following two steps: (1) title and abstract review and (2) full-text review. Discrepancies will be discussed to reach consensus where possible; otherwise, a third reviewer will resolve the dispute.

**Results:**

The results and a final report are expected to be completed and submitted to a peer-reviewed journal in 6 months.

**Conclusions:**

The review will examine existing literature to identify the impact of secure messaging in diabetes treatment within primary care settings. Research gaps will also be identified to determine if there is a need for further studies.

**International Registered Report Identifier (IRRID):**

DERR1-10.2196/42339

## Introduction

The prevalence of diabetes worldwide is expected to continue to rise. From 2010 to 2030, the prevalence of diabetes among individuals aged 20 to 79 years will likely increase from 6.4% to 7.7% worldwide and from 11.6% to 13.9% in Canada [1].

Diabetes is an endocrine disorder that arises from the body’s inability to produce insulin, effectively use it, or both, resulting in an unhealthy rise in blood sugar levels. Insulin is a hormone that is produced by the pancreas to regulate blood sugar. Consequences of unmanaged diabetes include pain, nerve damage, amputation, the failure of different organs, cardiovascular diseases, and more. Complications of this disease are very serious and can lead to death [2,3].

Canada’s public health care system spends billions of dollars annually in the treatment of chronic diseases, and this cost is rising due to population growth and improvements in screening, diagnosis, and care. Diabetes Canada estimated the direct cost of diabetes to the Canadian health care system to be about CAD $32 billion (approximately US $24 billion) in 2023 and predicted it to reach about CAD $39 billion (approximately US $29 billion) by 2028 [3]. The province of Alberta spends approximately twice as much money on patients with diabetes compared to the amount of money it spends on those without it [4].

The recent COVID-19 pandemic has acted as a catalyst for the adoption of virtual care technology and services around the world, including Canada. The Canadian government continues to invest in virtual care technology (eg, secure messaging technology, videoconferencing technology, and other tools), in addition to the billions of dollars already invested in digital health [5]. *Virtual care* is defined as “[a]ny interaction between patients and/or members of their circle of care occurring remotely, using any form of communication or information technology with the aim of facilitating or maximizing the quality of patient care.“ *Secure messaging* is a virtual care modality defined as “the asynchronous exchange of information between providers and patients, or between providers, through electronic platforms (e.g. texting, e-mail) that adhere to the standards of safety and privacy” [6]. Effective diabetes treatment involving the tracking and monitoring of diet, blood sugar, and physical activities that involve using technology together with active treatment is shown to be cost-effective in reducing complications. The provision of remote health care services by using a variety of virtual care tools is identified by Diabetes Canada as a delivery mechanism that can improve patient outcomes by providing timely and interdisciplinary care [3]. In contrast with other outcomes of conventional diabetes treatment, the use of virtual care offers benefits, such as improved access to services in rural or remote areas, reduced wait times, the avoidance of clinician and patient travel, cost efficiencies, and even reduced carbon emissions [3,7,8]. It is unclear to what extent the use of virtual care technology in health care helps to facilitate or maximize the quality of care for patients.

Our preliminary literature scan found many studies on the use of one or another form of digital health technology (telehealth, telemedicine, telemonitoring, videoconferencing, SMS text messages, electronic health records, etc) to improve diabetes management and patient outcomes, but only a small proportion are on the use of secure messaging for diabetes management. Similar findings were also suggested by a scoping review on the costs and benefits of eHealth applications that support the independent living of older adults [9]. A literature scan on virtual care evaluations, which was conducted by the Canadian Agency for Drugs and Technologies in Health in 2021, noted that studies tend to assess combined virtual care modalities (eg, video and telephone), making it difficult to provide informative evidence on the impact of a specific modality [10].

There is some evidence suggesting that the use of virtual care technology in the management of diabetes and other chronic diseases is associated with positive clinical outcomes that, in some cases, are comparable to those of in-person care [11-15]. Moreover, the use of asynchronous electronic communications tools is shown to improve primary care providers’ access to specialists, minimize unnecessary referrals, and reduce call volumes to providers [3,16-19]. However, negative impacts on clinicians' workloads and mixed inconclusive impacts on certain clinical outcomes (eg, blood pressure and cholesterol) and aspects of the quality of care have also been reported [11,15,19-22].

We find that literature on the impact of secure messaging in diabetes management tends to focus on discrete outcomes, such as biomedical outcomes, cost, adoption, patient satisfaction, and workflows, among others. We posit that there is a research gap on studies that assess the impact of secure messaging in primary care through a more holistic quality of care lens (ie, safety, effectiveness, efficiency, timeliness, and patient centricity, as defined by the Institute of Medicine [23]). A recent systematic review of patient portal functionalities and outcomes of patients with diabetes also identified similar gaps and the need for further studies to understand how the use of patient portals by patients with diabetes improves health outcomes [15].

To continue investing in virtual care services, health care policy makers need evidence on both the clinical outcomes and the health system outcomes to inform their decisions. The purpose of our scoping review is to explore what is known about the use of secure messaging in the treatment of diabetes within the primary care setting and how its impact has been assessed from the patient, health system, and quality of care perspectives.

Our scoping review will contribute to existing literature by examining the literature for evidence on the impact of secure messaging in the treatment of diabetes from a more holistic quality of care perspective that includes both clinical outcomes and health system outcomes.

## Methods

### Study Design

Our review will be based on the Arksey and O’Malley methodological framework for scoping reviews, which includes the following six steps: (1) identifying the research question; (2) identifying the relevant studies; (3) defining the study selection criteria; (4) charting the data; (5) collating, summarizing, and reporting the results; and (6) optionally consulting with peers for additional feedback and the validation of findings [24].

Our approach will result in the wide coverage of the literature. The decision to narrow down the literature will be made once the reviewers have a sense of the volume and general scope of the literature and gain familiarity with the literature and the field, after conducting the search [24]. The tools recommended by Levac et al [25] and the Joanna Briggs Institute [26-28], which are grounded on Arksey and O’Malley’s [24] earlier work, will be leveraged to conduct and report our scoping review in a structured way. These tools include the Population, Concept, and Context (PCC) framework and the PRISMA-ScR (Preferred Reporting Items for Systematic Reviews and Meta-Analyses Extension for Scoping Reviews) guidelines and checklist.

### Stage 1: Identifying the Research Question

Our scoping review aims to address the following research questions:

1. How is secure messaging used in the treatment of diabetes within the primary care setting?
2. Which outcome measures are used?
3. What is the impact of using secure messaging on the quality of care?
4. What are the facilitators, risks, and challenges of adopting secure messaging for diabetes care?

### Stage 2: Identifying Relevant Studies

The PCC framework was utilized to identify the main concepts of the research questions (Table 1). Initial iterative searches in MEDLINE were conducted by the principal investigator (AL) to identify relevant articles and subject headings.

**Table 1:** Population, Concept, and Context framework and search terms.

	Main concept keywords	Alternative keywords	Medical Subject Headings
Participants	*diabetic patients*	*Diabetes Mellitus*	*Diabetes Mellitus*
Concept	*secure message*	*secure messaging* *secure communication* *secure email* *virtual care* *virtual health* *telehealth* *telemedicine* *e-visits data* *mhealth* *patient portal*	*Electronic Health Records* *Communication* *Telemedicine* *Patient portal* *Personal Health Records* *Computerized Medical Records Systems* *Electronic Mail*
Context	*Primary healthcare setting*	*Primary care setting* *Community setting* *Outpatient setting*	*Primary Health Care* *Community Health Services* *Outpatient*

Further iterative searches against MEDLINE were conducted in collaboration with a professional information specialist, resulting in a more refined search strategy that produced 4972 results (Multimedia Appendix 1). This search strategy was then submitted to a second information specialist for a peer review assessment and the validation of the quality and comprehensiveness of the search strategy, in accordance with the PRESS (Peer Review of Electronic Search Strategies) 2015 guideline [29]. Comments were reviewed, and adjustments were made to the strategy as deemed appropriate.

The intervention of interest is secure messaging use in the treatment of diabetes within primary care settings. The care settings will comprise noninstitutionalized and nonhospital settings. This aligns with the definitions of *primary care* by the World Health Organization [30] and Health Canada, whereby primary care is considered an “element within primary health care that focusses on health care services, including health promotion, illness and injury prevention, and the diagnosis and treatment of illness and injury” [31].

Our strategy utilizes a combination of controlled vocabulary (eg, *Diabetes Mellitus*, *Telemedicine*, and *Patient Portals*) and free-text terms (eg, *NIDDM*, *virtual consult*, and *secure email*). Using the Ovid platform, we will search Ovid MEDLINE ALL, Embase, and APA PsycINFO. We will also search the Cochrane Library on Wiley, CINAHL on EBSCO, and PubMed. Vocabulary and syntax will be adjusted across the databases. No language or date limits will be applied, but animal-only records will be removed where possible. Results will be downloaded and deduplicated by using EndNote 9.3.3 (Clarivate Plc) and uploaded to Covidence (Veritas Health Innovation Ltd).

The reference lists of all included studies will also be hand searched to identify additional literature that may not be captured through the database search. Additional grey literature will also be identified through an existing network of reviewers, relevant journals, organizations, conferences, and a community of practices across the globe. EndNote software will be utilized to manage references.

### Stage 3: Defining the Study Selection Criteria

Based on the research questions and objectives, the inclusion and exclusion criteria shown in Table 2 will be used to initially guide the review. The further refinement of these criteria will occur in an iterative manner as the reviewers gain a better understanding of the scope of available literature. In alignment with the PCC framework, the initial inclusion criteria will include those for the population (patients with diabetes), concept (secure messages), and context (primary and community care settings). The use of secure messaging for patient-provider, caregiver-provider, and provider-provider communications will be included. The initial exclusion criteria will be patients with gestational diabetes, other virtual or digital health interventions, and institutionalized or hospital settings.

**Table 2:** Inclusion and exclusion criteria.

	Inclusion criteria	Exclusion criteria
Year of publication	No limit	N/A^a^
Language	English	All other languages
Population	Patients with diabetes (types 1 and 2)	Patients with gestational diabetes
Concept or intervention	Secure message	Other virtual care modalities (videoconferences and phone calls)
Context and setting	Primary health care setting Primary care setting Outpatient setting Community setting	Hospital setting Inpatient setting Institutionalized treatment within acute care setting
Study design	Studies that contain defined impacts and outcomes of secure messaging for diabetes treatment	Studies that do not contain defined impacts of secure messaging for diabetes treatment Commentaries Opinions Summary articles Clinical trial recruitments Scoping or systematic review protocols Abstracts
Outcomes	All patient-related clinical outcomes and health system outcomes (eg, patient experience, patient clinical outcomes, health system outcomes, health care utilization, quality of life outcomes, health status, emergency room visits, follow-up visits, etc)	Nonclinical outcomes and outcomes unrelated to health systems

^a^N/A: not applicable.

### Stage 4: Charting the Data

The review team consists of the following three reviewers: the principal investigator (AL), the supervisor (DM), and the cosupervisor (TS). Due to its convenience, Covidence will be used by the reviewers to independently screen the articles, based on the inclusion and exclusion criteria, in the following two steps: (1) title and abstract review and (2) full-text review of the full-text articles and documents. To be more inclusive and reduce the risk of excluding relevant studies, any articles that reviewers are unsure of during the title and abstract scan will be moved forward to the full-text review stage.

Data extraction will be conducted outside of Covidence, using a charting form (Multimedia Appendix 2) that we initially created based on Arksey and O’Malley’s [24] recommendations, which include the following elements: author(s), year of publication, study location, intervention type, comparator (if any), duration of the intervention, study populations, aims of the study, methodology, outcome measures, and important results.

Two reviewers will pilot the data extraction form on the first 30 articles to identify the need for modification. The review team will further revise the form, as needed, during the data extraction process as new relevant data types and themes are encountered.

### Stage 5: Collating, Summarizing, and Reporting the Results

The review team will utilize the PRISMA (Preferred Reporting Items for Systematic Reviews and Meta-Analyses) chart and PRISMA-ScR checklist [27] to report their findings in a clear, transparent way (Multimedia Appendix 3 [28] and Multimedia Appendix 4 [27]).

A narrative summary of findings will be developed based on the elements of the data extraction form and guided by the context of the research question and study objectives. Throughout the course of the review, if other criteria or elements are uncovered, the authors will decide on other additional formats (eg, graphs, tables, and diagrams) for presenting the study findings.

### Stage 6: Peer Consultation for Feedback and Validation

At different stages, experts will be consulted to review and provide feedback. In stage 2, prior to executing the final search, the search strategy will undergo a peer review by a qualified librarian to ensure relevance and achieve more holistic search results. Other experts within the field will also be drawn from our networks to ensure that relevant findings are captured and reported in a clear manner. In stages 3 to 5, experts will also be consulted to suggest additional literature and provide feedback on the data extraction form.

The experts we consult will include researchers and practitioners within the digital health field and decision makers from publicly funded payer organizations.

### Ethical Considerations

Neither ethics approval nor participant consent is required, as our scoping review will not include human or animal participants. Data will be collected from published literature.

## Results

The systematic literature search of databases has been conducted, and the title and abstract review is planned to commence in early September 2022.

An article of the scoping review findings will be submitted for publication in a peer-reviewed journal. The final version of findings and the full-text article will be shared with a panel and wider stakeholder organizations.

## Discussion

### Overview

Our scoping review will explore what is known about the use of secure messaging in the treatment of diabetes within the primary care setting. The review will contribute to knowledge on this topic by examining how the impact of secure messaging has been assessed from the patient, health system, and quality of care perspectives. To our knowledge, this has not been the focus of previous reviews [11,15,22,23].

The findings of the scoping review will inform health care policy makers’ decisions on virtual care services.

### Strengths and Limitations

Our scoping review will use an established framework and a comprehensive systematic search to identify and review existing literature on the use of secure messaging in the treatment of diabetes to date. The search strategy is peer-reviewed, and the grey literature search and the review will be informed and guided via consultation with experts. Although the findings will be useful in understanding the state of the literature and potential research gaps, the study does not aim to conduct a full assessment of the quality of the evidence in the literature uncovered.

## Appendix

Multimedia Appendix 1 Search strategy.

Multimedia Appendix 2 Data extraction form.

Multimedia Appendix 3 PRISMA (Preferred Reporting Items for Systematic Reviews and Meta-Analyses) 2020 flow diagram template [28].

Multimedia Appendix 4 PRISMA-ScR (Preferred Reporting Items for Systematic Reviews and Meta-Analyses Extension for Scoping Reviews) checklist [27].

## Abbreviations

PCC: Population, Concept, and Context
PRESS: Peer Review of Electronic Search Strategies
PRISMA: Preferred Reporting Items for Systematic Reviews and Meta-Analyses
PRISMA-ScR: Preferred Reporting Items for Systematic Reviews and Meta-Analyses Extension for Scoping Reviews

## References

[1] Shaw JE, Sicree RA, Zimmet PZ (2010). Global estimates of the prevalence of diabetes for 2010 and 2030. Diabetes Res Clin Pract.

[2] American Diabetes Association (2014). Diagnosis and classification of diabetes mellitus. Diabetes Care.

[3] Diabetes Canada (2018). Diabetes 360º: A framework for a diabetes strategy for Canada. Diabetes Canada.

[4] Government of Alberta (2013). Health care system cost of people with diabetes. Government of Alberta.

[5] Government of Canada Pan-Canadian virtual care priorities in response to COVID-19. Government of Canada.

[6] Task Team on Equitable Access to Virtual Care (2021). Enhancing equitable access to virtual care in Canada: Principle-based recommendations for equity. Health Canada.

[7] Hackett C, Brennan K, Fowler HS, Leaver C (2019). Valuing citizen access to digital health services: Applied value-based outcomes in the Canadian context and tools for modernizing health systems. J Med Internet Res.

[8] Shimada SL, Zocchi MS, Hogan TP, Kertesz SG, Rotondi AJ, Butler JM, Knight SJ, DeLaughter K, Kleinberg F, Nicklas J, Nazi KM, Houston TK (2020). Impact of patient-clinical team secure messaging on communication patterns and patient experience: Randomized encouragement design trial. J Med Internet Res.

[9] Sülz S, van Elten HJ, Askari M, Weggelaar-Jansen AM, Huijsman R (2021). eHealth applications to support independent living of older persons: Scoping review of costs and benefits identified in economic evaluations. J Med Internet Res.

[10] Hui D, Dolcine B, Loshak H (2022). Approaches to evaluations of virtual care in primary care. Canadian Journal of Health Technologies.

[11] Nguyen OT, Tabriz AA, Huo J, Hanna K, Shea CM, Turner K (2021). Impact of asynchronous electronic communication-based visits on clinical outcomes and health care delivery: Systematic review. J Med Internet Res.

[12] Hawes EM, Lambert E, Reid A, Tong G, Gwynne M (2018). Implementation and evaluation of a pharmacist-led electronic visit program for diabetes and anticoagulation care in a patient-centered medical home. Am J Health Syst Pharm.

[13] Robinson SA, Netherton D, Zocchi M, Purington C, Ash AS, Shimada SL (2021). Differences in secure messaging, self-management, and glycemic control between rural and urban patients: Secondary data analysis. JMIR Diabetes.

[14] Shimada SL, Allison JJ, Rosen AK, Feng H, Houston TK (2016). Sustained use of patient portal features and improvements in diabetes physiological measures. J Med Internet Res.

[15] Alturkistani A, Qavi A, Anyanwu PE, Greenfield G, Greaves F, Costelloe C (2020). Patient portal functionalities and patient outcomes among patients with diabetes: Systematic review. J Med Internet Res.

[16] Liddy C, Afkham A, Drosinis P, Joschko J, Keely E (2015). Impact of and satisfaction with a new eConsult service: A mixed methods study of primary care providers. J Am Board Fam Med.

[17] Canada Health Infoway (2020). Analysis of the current and potential benefits of virtual care in Canada. Canada Health Infoway.

[18] Huang M, Fan J, Prigge J, Shah ND, Costello BA, Yao L (2022). Characterizing patient-clinician communication in secure medical messages: Retrospective study. J Med Internet Res.

[19] Hoonakker PLT, Carayon P, Cartmill RS (2017). The impact of secure messaging on workflow in primary care: Results of a multiple-case, multiple-method study. Int J Med Inform.

[20] Heisey-Grove DM, McClelland LE, Rathert C, Tartaglia A, Jackson K, DeShazo JP (2020). Associations between patient health outcomes and secure message content exchanged between patients and clinicians: Retrospective cohort study. J Med Internet Res.

[21] Heisey-Grove D, Rathert C, McClelland LE, Jackson K, DeShazo JP (2021). Patient and clinician characteristics associated with secure message content: Retrospective cohort study. J Med Internet Res.

[22] Kuo A, Dang S (2016). Secure messaging in electronic health records and its impact on diabetes clinical outcomes: A systematic review. Telemed J E Health.

[23] Institute of Medicine, Committee on Quality of Health Care in America (2001). Crossing the Quality Chasm: A New Health System for the 21st Century.

[24] Arksey H, O'Malley L (2005). Scoping studies: towards a methodological framework. Int J Soc Res Methodol.

[25] Levac D, Colquhoun H, O'Brien KK (2010). Scoping studies: advancing the methodology. Implement Sci.

[26] Peters MDJ, Godfrey CM, Khalil H, McInerney P, Parker D, Soares CB (2015). Guidance for conducting systematic scoping reviews. Int J Evid Based Healthc.

[27] Tricco AC, Lillie E, Zarin W, O'Brien KK, Colquhoun H, Levac D, Moher D, Peters MDJ, Horsley T, Weeks L, Hempel S, Akl EA, Chang C, McGowan J, Stewart L, Hartling L, Aldcroft A, Wilson MG, Garritty C, Lewin S, Godfrey CM, Macdonald MT, Langlois EV, Soares-Weiser K, Moriarty J, Clifford T, Tunçalp Ö, Straus SE (2018). PRISMA Extension for Scoping Reviews (PRISMA-ScR): Checklist and explanation. Ann Intern Med.

[28] Page MJ, McKenzie JE, Bossuyt PM, Boutron I, Hoffmann TC, Mulrow CD, Shamseer L, Tetzlaff JM, Akl EA, Brennan SE, Chou R, Glanville J, Grimshaw JM, Hróbjartsson A, Lalu MM, Li T, Loder EW, Mayo-Wilson E, McDonald S, McGuinness LA, Stewart LA, Thomas J, Tricco AC, Welch VA, Whiting P, Moher D (2021). The PRISMA 2020 statement: an updated guideline for reporting systematic reviews. BMJ.

[29] McGowan J, Sampson M, Salzwedel DM, Cogo E, Foerster V, Lefebvre C (2016). PRESS Peer Review of Electronic Search Strategies: 2015 guideline statement. J Clin Epidemiol.

[30] World Health Organization Primary care. World Health Organization.

[31] Government of Canada About primary health care. Government of Canada.

