# Supplementary material for: The Impact of Secure Messaging in the Treatment of Patients With Diabetes Within a Primary Care Setting: Protocol for a Scoping Review
Source: JMIR Res Protoc. 2023 May 2;12:e42339. doi: 10.2196/42339 (PMC10189617; doi:10.2196/42339)
Supplement: Multimedia Appendix 1 [file resprot_v12i1e42339_app1.docx]

Search Strategy

| Database: Ovid MEDLINE(R) ALL <1946 to May 18, 2022>  Search Strategy:  --------------------------------------------------------------------------------  1 Diabetes Mellitus/ (130414)  2 exp Diabetes Mellitus, Type 1/ (83026)  3 exp Diabetes Mellitus, Type 2/ (157617)  4 diabet*.tw,kw,kf. (723446)  5 (DM adj2 (insulin dependen* or insulin independen* or non-insulin dependen* or noninsulin dependen* or early onset or adult onset or ketoacido* or ketos#s resist#n* or labile or maturity onset or juvenile or brittle or sudden-onset or type 1 or type I or type 2 or type II)).tw,kw,kf. (6083)  6 (DM1 or "DM 1" or "DM T1" or IDDM or ID DM or T1D or T1DM or T1 DM or mckusick 22210).tw,kw,kf. (24857)  7 (DM2 or "DM 2" or "DM T2" or MODY or NIDDM or NID DM or T2D or T2DM or T2 DM).tw,kw,kf. (52625)  8 or/1-7 [DM] (767805)  9 *Telemedicine/ (28272)  10 (telemed* or tele-med* or telecare or tele-care or telecoach* or tele-coach* or teleconsult* or tele-consult* or telehealth* or tele-health* or telemonitor* or tele-monitor* or telesupport* or tele-support* or teletherap* or tele-therap* or teletreatment* or tele-treatment*).ti,kw,kf. (23898)  11 (ehealth* or e-health* or mhealth* or m-health*or ecoach* or e-coach* or mcoach* or m-coach* or esupport* or e-support* or msupport* or m-support* or etherap* or e-therap* or mtherap* or m-therap* or evisit* or e-visit* or mvisit* or m-visit*).ti,kw,kf. (12790)  12 (emedicine* or e-medicine*).ti,kw,kf. (47)  13 (mobile health* or mobile care or mobile medicine).ti,kw,kf. (4341)  14 ((digital* or virtual* or remote*) adj3 (care or health* or healthcare or health-care)).ti,kw,kf. (7327)  15 ((digital* or virtual* or remote*) adj3 (appointment* or clinic or clinics or communicat* or consult* or followup or follow-up or hub or hubs or interven* or manag* or meet* or monitor* or support* or therap* or tool or tools or treatment? or visit*)).ti,kw,kf. (11524)  16 (e-provider? or e-clinician? or e-doctor? or e-nurse? or e-physician? or e-practitioner? or e-therapist? or m-provider? or m-clinician? or m-doctor? or m-nurse? or m-physician? or m-practitioner? or m-therapist?).ti,kw,kf. (13)  17 Electronic Mail/ (2893)  18 Patient Portals/ (654)  19 (patient? adj2 (portal or portals)).tw,kw,kf. (5394)  20 ((web or web-based or web-site or website or internet or online or www or cyber*) adj3 (communicat* or messag* or portal or portals)).tw,kw,kf. (5649)  21 (secure* adj3 (communicat* or electronic mail* or email* or e-mail* or messag* or platform* or portal or portals)).tw,kw,kf. (1678)  22 ((e-mail* or email* or electronic mail* or online messag* or instant* messag* or textmessag* or text-messag*) adj3 (care or health care or health-care or healthcare or appointment* or conferenc* or consult* or interven* or manag* or meet* or monitor* or support* or therap* or treatment? or visit*)).tw,kw,kf. (2902)  23 ((e-mail* or email* or electronic mail* or online messag* or instant* messag* or textmessag* or text-messag*) adj3 (clinician* or doctor* or nurse* or physician* or practitioner* or therapist*)).tw,kw,kf. (757)  24 (e-mail* or email* or electronic mail* or online messag* or instant* messag* or textmessag* or text-messag*).ti,kw,kf. (3915)  25 (e-mail* or email* or electronic mail* or online messag* or instant* messag* or textmessag* or text-messag*).ab. /freq=2 (6011)  26 *Electronic Health Records/ (15169)  27 ((health record? or EHR or EHRs or PHR or PHRs) adj3 (portal or portals)).tw,kw,kf. (220)  28 ((medical record? or EMR or EMRs or PMR or PMRs) adj3 (portal or portals)).tw,kw,kf. (51)  29 ((health information or medical information or clinical information) adj3 (portal or portals)).tw,kw,kf. (91)  30 ((health data or medical data or clinical data) adj3 (portal or portals)).tw,kw,kf. (42)  31 ((health or healthcare or health care) adj portal?).tw,kw,kf. (182)  32 ((ehealth or e-health or mhealth or m-health or mobile health) adj3 (portal or portals)).tw,kw,kf. (55)  33 or/9-32 [SECURE MESSAGING/PATIENT PORTALS PT 1] (94976)  34 Medical Records Systems, Computerized/ (19135)  35 Electronic Health Records/ (25133)  36 Health Records, Personal/ (1691)  37 (health record? or EHR or EHRs or PHR or PHRs or medical record? or EMR or PMR or EMR or EMRs).ti,kw,kf. (26187)  38 (health record? or EHR or EHRs or PHR or PHRs or medical record? or EMR or PMR or EMR or EMRs).ab. /freq=3 (18110)  39 or/34-38 [EHR/PHR] (63716)  40 Internet/ (79156)  41 Patient Participation/ (28556)  42 ((patient or patients) adj3 (activat* or communicat* or empower* or engag* or participat*)).tw,kw,kf. (82176)  43 Self Care/ (35297)  44 Self-Management/ (4519)  45 or/40-44 [PATIENT ENGAGEMENT] (216289)  46 39 and 45 [PATIENT PORTALS, PT 2] (4581)  47 Professional-Patient Relations/ or Physician-Patient Relations/ or Nurse-Patient |
| --- |
