# Supplementary material for: The Impact of Secure Messaging in the Treatment of Patients With Diabetes Within a Primary Care Setting: Protocol for a Scoping Review
Source: JMIR Res Protoc. 2023 May 2;12:e42339. doi: 10.2196/42339 (PMC10189617; doi:10.2196/42339)
Supplement: Multimedia Appendix 2 [file resprot_v12i1e42339_app2.docx]

|  | Description |
| --- | --- |
|  |  |
| **Author(s)** |  |
| **Year of Publication** |  |
| **Study Location (Country)** |  |
| **Aim of Study** |  |
| **Study Type** |  |
| **Methodology** |  |
| **Setting** |  |
| **Provider** | physician, nurse, admin, specialist, other? |
| **Population Characteristics** |  |
| **Disease (Type I, Type II, or both?)** |  |
| **Intervention**   - Type (patient-provider, caregiver-provider, provider-provider) - How intervention is used |  |
| **Comparator (if any)** |  |
| **Duration of Intervention** |  |
| **Outcome Measures:**   - Patient/caregiver experience - Provider experience - Population health - Cost/efficiency - Safety - Timeliness - Equity - Quality of life - Healthcare utilization |  |
| **Other Important Results** |  |
|  |  |

Data Extraction Form
